# Effect of Acidic Environment on the Mechanical Strength and Surface Properties of Three-Dimensional-Printed Denture Base Resin

**DOI:** 10.1016/j.identj.2026.109757

**Published:** 2026-07-17

**Authors:** Eun-Ju Kim, Se-Eun Kim, Ye-Jin Kim, Ji-Eun Kim, Yun-Yeong Hwang, Hye-Bin Go, Song-Yi Yang

**Affiliations:** a Department of Dental Hygiene, Konyang University, Daejeon, Republic of Korea; b Department and Research Institute of Dental Biomaterials and Bioengineering, Yonsei University College of Dentistry, Seoul, Republic of Korea

**Keywords:** 3D printing, Additive manufacturing, Acidic environment, Denture base resin, Mechanical properties, Surface roughness

## Abstract

**Introduction and aims:**

This study evaluated the effects of acidic immersion conditions on the mechanical properties, colour stability, microhardness, and surface roughness of a three-dimensional (3D) printed denture base resin compared with a conventional autopolymerized resin.

**Methods:**

Specimens of both materials were immersed for 24 hours at 36°C in solutions with pH values of 1, 3, 5, or 7. Flexural strength and modulus were measured using a three-point bending test, and Vickers hardness, colour stability, surface roughness, and contact angle were evaluated using established laboratory protocols. Surface morphology was examined using scanning electron microscopy to assess microstructural changes under different pH conditions.

**Results:**

Two-way ANOVA showed that material type and pH level significantly affected flexural strength, flexural modulus, and Vickers hardness (*P* < .05), with significant material × pH interactions for flexural modulus and Vickers hardness. The tested 3D-printed resin generally showed higher mechanical properties than the autopolymerized resin under the tested pH conditions. Surface roughness was significantly affected by pH level and increased under lower pH conditions, while scanning electron microscopy qualitatively showed more visible surface irregularities at lower pH values. Colour stability and contact angle were not significantly affected by material type, pH level, or their interaction (*P* > .05).

**Conclusion:**

The tested 3D-printed denture base resin showed more favourable mechanical behaviour than the tested autopolymerized resin under the present acidic conditions. Further studies using broader material formulations and clinically relevant ageing models are needed to validate these findings.

## Introduction

For decades, dentures have played an essential functional and aesthetic role in restoring oral function in patients with edentulism. Polymethyl methacrylate (PMMA) has long been the primary material used in denture fabrication because of its aesthetic appeal, ease of processing, low cost, and simple maintenance in clinical settings.^1^ Despite these advantages, PMMA has inherent limitations, including susceptibility to fracture, reduced mechanical integrity under prolonged chemical exposure, and potential allergenic responses, all of which may compromise its clinical reliability and longevity as a denture base material.^2^

To overcome these limitations, advances in digital dentistry have introduced additive manufacturing, commonly known as three-dimensional (3D) printing, as a novel approach for denture fabrication. Among the various additive manufacturing techniques, digital light processing (DLP) uses ultraviolet or visible light-induced photopolymerization to produce highly precise, layer-by-layer polymerized prosthetic components.^2^^,^^3^ In dentistry, 3D printing is used in several fields, including prosthodontics, oral and craniofacial surgery, orthodontics, periodontics, endodontics, and oral implantology.^4^ Compared to conventionally manufactured dentures, 3D printing technology offers advantages such as fewer clinical visits for patients and improved biocompatibility.^5^ However, because 3D-printed dentures are relatively new in clinical practice, it remains essential to thoroughly evaluate their mechanical performance and surface stability under diverse oral environments.

The oral cavity frequently experiences fluctuations in acidity influenced by dietary habits, beverage consumption, medication use, and pathological conditions such as gastroesophageal reflux disease.^6^ Many acidic beverages, including carbonated soft drinks, sports drinks, and citrus-based products, contain strong or weak acids (eg, hydrochloric, phosphoric, or citric acids) that can lower intraoral pH and adversely affect the surface and mechanical properties of dental materials. Such fluctuations in acidity may be influenced by bacterial acid production and other intraoral factors, potentially contributing to changes in denture base resin properties.^7^ Previous studies have demonstrated that prolonged exposure to acidic environments can adversely affect the structural integrity and surface characteristics of conventional denture base materials and computer-aided design/computer-aided manufacturing restorations.^8^^,^^9^ Furthermore, differences were observed in the viability and physical properties of cells in the oral cavity under neutral and alkaline conditions, and a general decline in performance was observed in the oral cavity.^10^

Despite growing clinical interest in 3D-printed resins, limited research has evaluated their resistance to acidic environments that commonly occur in daily life. Acid exposure may influence the flexural strength, hardness, colour stability, and surface morphology of 3D-printed materials differently from conventional PMMA because of differences in polymerization, cross-linking density, and resin composition. Furthermore, because patient expectations for aesthetics continue to increase, understanding the colour stability and surface characteristics of denture materials under low-pH conditions is clinically important.^11^, ^12^, ^13^ In this study, an autopolymerized resin was selected as the control group. While heat-polymerized PMMA is often regarded as the gold standard for definitive dentures, autopolymerized resin was chosen to provide a clinical benchmark for materials typically used in provisional and repair applications, where 3D-printing technology is increasingly being implemented for its time efficiency.

**Fig. 1:**
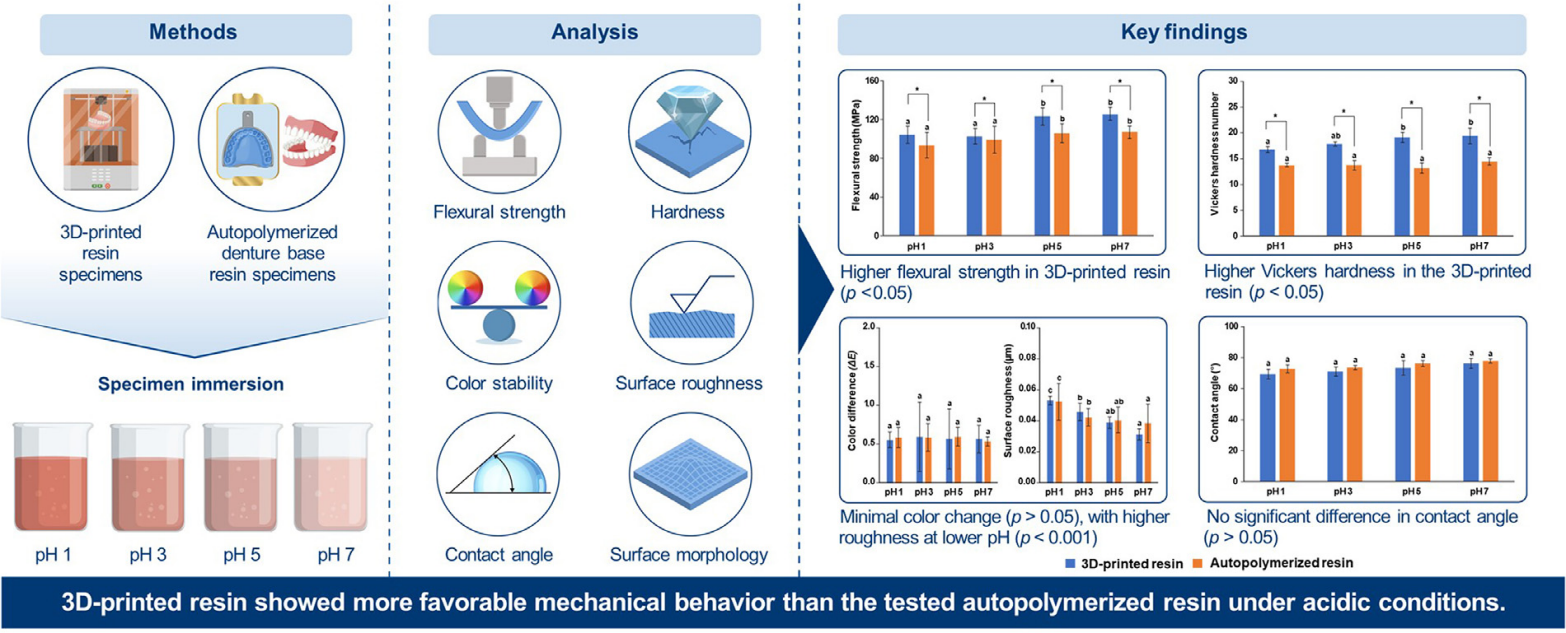
Graphical abstract of the study. This diagram illustrates the comparison between 3D-printed and conventional autopolymerized resins across various pH levels (1-7).

Therefore, this study aimed to evaluate and compare the flexural strength, Vickers hardness, colour stability, surface roughness, contact angle, and surface morphology of 3D-printed and autopolymerized denture base resins after immersion in acidic solutions of varying pH (Figure 1). The null hypothesis was that acidic environments would not significantly affect the mechanical or surface properties of either denture base resin.

## Materials and methods

### Specimen preparation

The following two types of denture base resins were used in this study: a 3D-printed resin (NextDent, Vertex Dental BV) and a conventional autopolymerized denture base resin (ProBase Cold, Ivoclar Vivadent). In total, 64 specimens (32 per resin type) were prepared in bar-shaped dimensions of 64.0 mm (length) × 10.0 mm (width) × 3.3 mm (thickness) (±0.2 mm), following ISO 20795-1:2013 (Dentistry – Base polymers – Part 1: Denture base polymers) standards.^14^ The dimensions of all specimens were verified using a digital calliper before testing to ensure compliance with the ISO standard. Each resin group was further subdivided into four pH subgroups (pH 1, 3, 5, and 7), with eight specimens in each subgroup (*n* = 8). No additional subgroups were tested. The sample size was selected based on previous studies with comparable experimental designs using similar group sizes.

#### 3D-printed resin specimens

Specimens were designed digitally and fabricated using a DLP 3D printer (NextDent 5100, Vertex Dental BV) with a layer thickness of 50 µm and a build orientation of 0°. After printing, the specimens were detached from the build platform, and the support structures were carefully removed. The specimens were cleaned with isopropyl alcohol using an ultrasonic cleaner (SH-2100, Saehan Ultrasonic Industry). After cleaning, an additional postcuring process was performed for 30 minutes in a postcuring oven (LC-3DPrint Box, NextDent, 3D Systems, Vertex Dental BV). Finally, the specimens were polished using a polishing machine (Ecomet, Buehler Ltd.) with silicon carbide paper (Deerfos) in the following grit sequences: 800, 1000, 1500, and 2000.

#### Autopolymerized resin specimens

Specimens were fabricated using ProBase Cold resin (Ivoclar Vivadent) by mixing 6 g of powder with 4 mL of liquid, according to the manufacturer’s guidelines. The mixed resin was poured into rectangular moulds with dimensions of 64.0 mm (length) × 10.0 mm (width) × 3.3 mm (thickness) (±0.2 mm), degassed for 15 minutes using an Auto Air Press Unit (Sejong Dental), and polymerized at room temperature (23 ± 2°C). After polymerization, the specimens were polished using a polishing machine with silicon carbide paper in the following grit sequences: 800, 1000, 1500, and 2000. The same polishing protocol was applied to both resin types to minimize surface preparation bias.

### Solution preparation

Acidic solutions at pH 1, 3, and 5 were prepared by diluting hydrochloric acid with distilled water. The neutral solution (pH 7) comprised sterile distilled water. All solutions were freshly prepared prior to specimen immersion, and the precise pH of each solution was measured using a calibrated pH meter (Orion Star A211; Thermo Scientific).

### Specimen immersion protocol

Hydrochloric acid (HCl) was selected as the immersion medium to represent a severe endogenous acidic challenge associated with gastroesophageal reflux disease.^15^^,^^16^ Specifically, the pH 1 group was included to model an extreme acidic condition, reflecting the intermittent exposure of denture base materials to gastric fluids with a pH of approximately 1.0 to 2.0 during severe reflux episodes.^11^ The 3D-printed and autopolymerized specimens were divided into four groups of eight specimens each (pH 1, 3, 5, and 7 solutions) in 50 mL conical tubes, and 25 mL of the pH solution was added to each group using a pipette aid. The specimens were maintained at 36°C for 24 hours in an incubated shaker (IST-4075R; Jeiotech) at 120 rpm. The specimens were then cleaned in distilled water using an ultrasonic cleaner (SH-2100, Saehan Ultrasonics) for 5 minutes to eliminate solution residues.

### Flexural strength

Eight specimens from each group were tested for three-point flexural strength using a universal testing machine (5942; Instron) under ambient laboratory conditions (23 ± 2°C, 50 ± 10% relative humidity). The length of the support was set to 50.0 mm, and the speed of the crosshead was set to 5.0 mm/min to apply a load at the midpoint of the specimen. Flexural strength and flexural modulus were calculated according to ISO 20795-1 using the following equations: The flexural strength, *σ*, and flexural modulus, *E*, were calculated using Equations (1) and (2), respectively.

(1) $$\sigma =\frac{3Fl}{2bh^{2}}$$

(2) $$E=\frac{Pl^{3}}{4bh^{3}d}$$

where *F* is the maximum load exerted on the specimen (N), *b* is the specimen width (mm), *h* is the specimen height (mm) measured immediately before the test, *P* is the load at a point in the linear region of the load-displacement curve (N), and *d* is the deflection at load *P* (mm).

### Vickers hardness

Surface microhardness was measured using a Vickers hardness tester (MMT-X; Matuzawa) with a load of 300 gf and a holding time of 30 seconds. The average value of the results obtained from three selected areas of each specimen was calculated and used as a representative value. Vickers hardness (VH) was calculated using the following equation:

(3) $$VH=\frac{1.854F}{d^{2}}$$

where *F* is the applied load (kgf), and *d* is the mean diagonal length of the indentation (mm).

### Colour stability

The CIELAB coordinates of the specimens were measured using the SCE method using a spectrophotometer (CM-5; Konica Minolta). All the specimens were dried in air for 10 seconds before measurement. Colour data were collected before and after immersion, and three randomized areas per specimen were measured, with the mean values recorded as representative data. The CIELAB colour difference (ΔE*) was calculated to allow direct comparison with previous studies evaluating colour stability of denture base resins under acidic conditions, using Equation (4).

(4) $$\Delta E^{*}=\;\sqrt{\left\{{\left(L_{2}^{*}-\;L_{1}^{*}\right)}^{2}+\;{\left(a_{2}^{*}-\;a_{1}^{*}\right)}^{2}+\;{\left(b_{2}^{*}-\;b_{1}^{*}\right)}^{2}\right\}}$$

where L* is the lightness, a* is the green-red axis, and b* is the blue-yellow axis. L*_1_, a*_1_*_,_* and b*_1_ are the CIELAB values of the sample before immersion, and L*_2_, a*_2_*_,_* and b*_2_ are the CIELAB values of the sample after immersion.

### Surface roughness

Surface roughness was measured using a contact stylus profilometer (DektakXT Stylus Profiler, Bruker) equipped with a stylus tip radius of 2 µm and a contact force of 0.7 mN. The probe traversed a 4.0 mm length at the centre of each specimen at a speed of 0.5 mm/s, with a cutoff value of 0.08 mm. Three measurements were performed for each specimen in different directions on the specimen surface to reduce directional measurement bias, and the average value was recorded as the final surface roughness.

### Contact angle

The contact angles were measured using a droplet analysis device (SmartDrop; FemtoFab). Using the sessile drop method, a 5 µL droplet of distilled water was dropped onto the surface, and the formed contact angle was measured at room temperature. Three measurements were taken for each specimen, and the average value was used for statistical analysis.

### Surface morphology

The surface morphologies of the specimens were examined using field-emission scanning electron microscopy (SEM; Carl Zeiss) at a magnification of 5000×. Before imaging, the specimens were sputter-coated with gold to enhance their conductivity. The surface features were qualitatively evaluated based on the SEM micrographs.

### Statistical analysis

The number of specimens per group was determined based on previous *in vitro* studies on dental polymer-based materials that used comparable experimental designs and similar group sizes. Statistical analyses were performed using IBM SPSS Statistics software (version 31.0; IBM Corp.). The assumptions required for parametric analyses were assessed before statistical testing. Two-way analysis of variance (ANOVA) was performed to evaluate the main effects of material type and pH level, as well as their interaction effect (material × pH), on the measured outcome variables. When significant main or interaction effects were detected, Tukey’s HSD posthoc test was performed for multiple comparisons. The significance level was set at *P* < .05.

## Results

### Flexural strength and flexural modulus

**Fig. 2:**
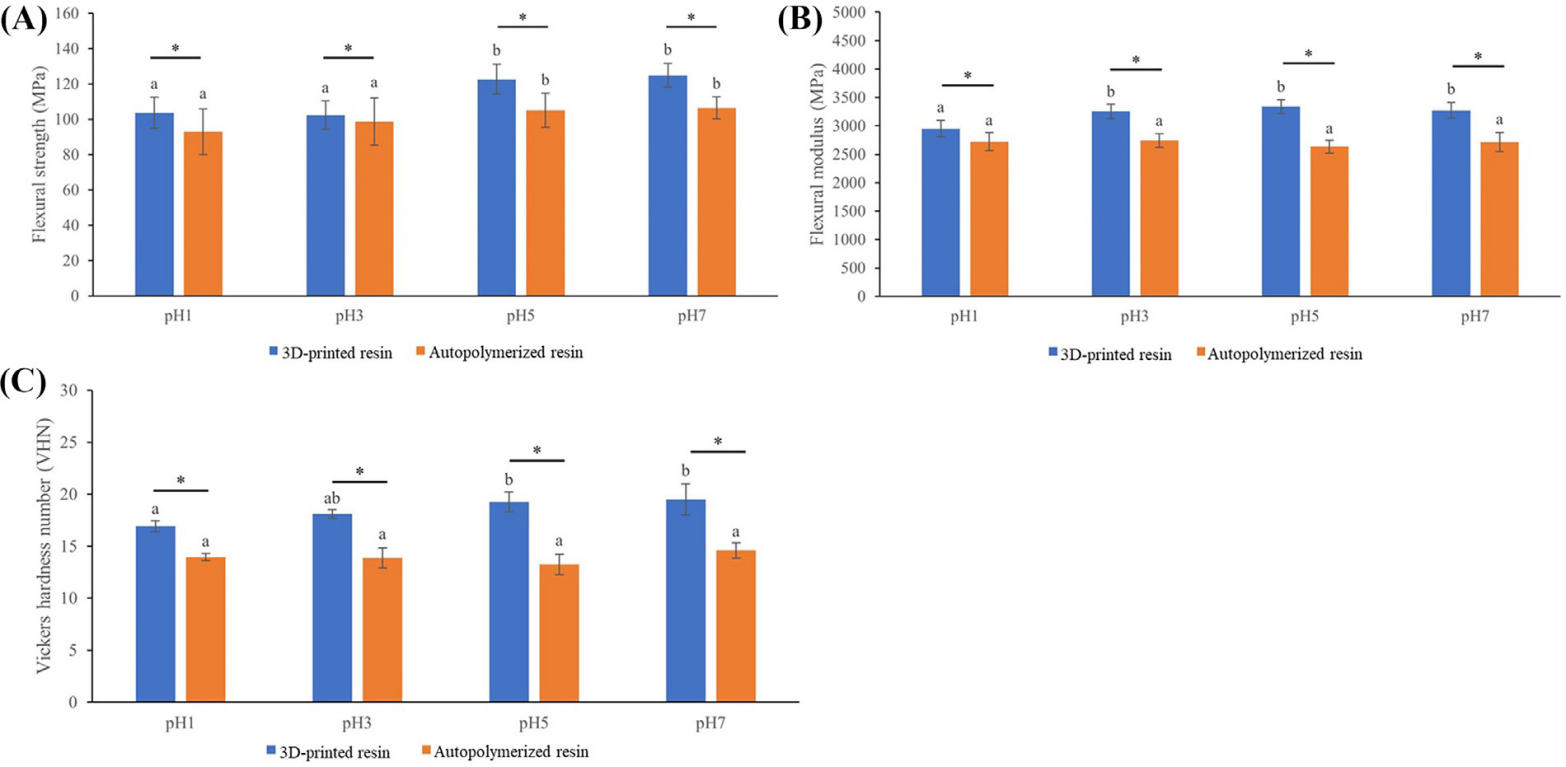
Three-dimensional (3D)-printed denture base resin and autopolymerized resin by pH: (A) flexural strength, (B) flexural modulus, and (C) Vickers hardness. Different lowercase letters within the same material indicate significant differences among pH groups based on posthoc comparisons (*P* < .05). An asterisk (*) indicates a significant difference between the 3D-printed denture base resin and autopolymerized resin within the same pH group (*P* < .05).

Regarding flexural strength, two-way ANOVA revealed significant main effects for both material type (*F* = 19.169, *P* < .001) and pH level (*F* = 9.675, *P* < .001), whereas the interaction effect (material × pH) was not statistically significant (*F* = 1.481, *P* = .236). These results indicate that flexural strength was significantly affected by material type and pH level, but the pH-related change did not significantly differ between the two resin types. As shown in Figure 2A, both the 3D-printed resin and the autopolymerized resin showed higher flexural strength values at pH 5 and pH 7 than at pH 1 and pH 3 (*P* < .05). Between-material comparisons showed that the 3D-printed resin had significantly higher flexural strength than the autopolymerized resin across all pH levels (*P* < .05).

For flexural modulus, two-way ANOVA demonstrated significant main effects for both material type (*F* = 115.818, *P* < .001) and pH level (*F* = 3.027, *P* = .045). A statistically significant interaction effect (material × pH) was also observed (*F* = 4.770, *P* = .008), indicating that the effect of pH on flexural modulus differed depending on the resin type. Posthoc analysis revealed that the autopolymerized resin exhibited no significant variation in flexural modulus across different pH conditions (*P* > .05). In contrast, the 3D-printed resin showed significantly higher flexural modulus values at pH 3, pH 5, and pH 7 than at pH 1 (*P* < .05). Between-material comparisons showed that the 3D-printed resin consistently exhibited higher flexural modulus values than the autopolymerized resin at all pH levels (*P* < .05) (Figure 2B).

### Vickers hardness

Regarding Vickers hardness, two-way ANOVA revealed significant main effects for both material type (*F* = 243.245, *P* < .001) and pH level (*F* = 4.890, *P* = .007). A statistically significant interaction effect (material × pH) was also observed (*F* = 4.866, *P* = .008), indicating that the effect of pH on Vickers hardness differed depending on the resin type. The Vickers hardness values are presented in Figure 2C. Posthoc analysis indicated that, in the 3D-printed resin group, hardness values were significantly higher at pH 5 and pH 7 than at pH 1 (*P* < .05), whereas pH 3 showed an intermediate value. In contrast, the autopolymerized resin showed no significant differences in hardness among pH levels (*P* > .05). Between-material comparisons showed that the 3D-printed resin exhibited significantly higher hardness values than the autopolymerized resin across all evaluated pH conditions (*P* < .05).

### Colour stability

**Fig. 3:**
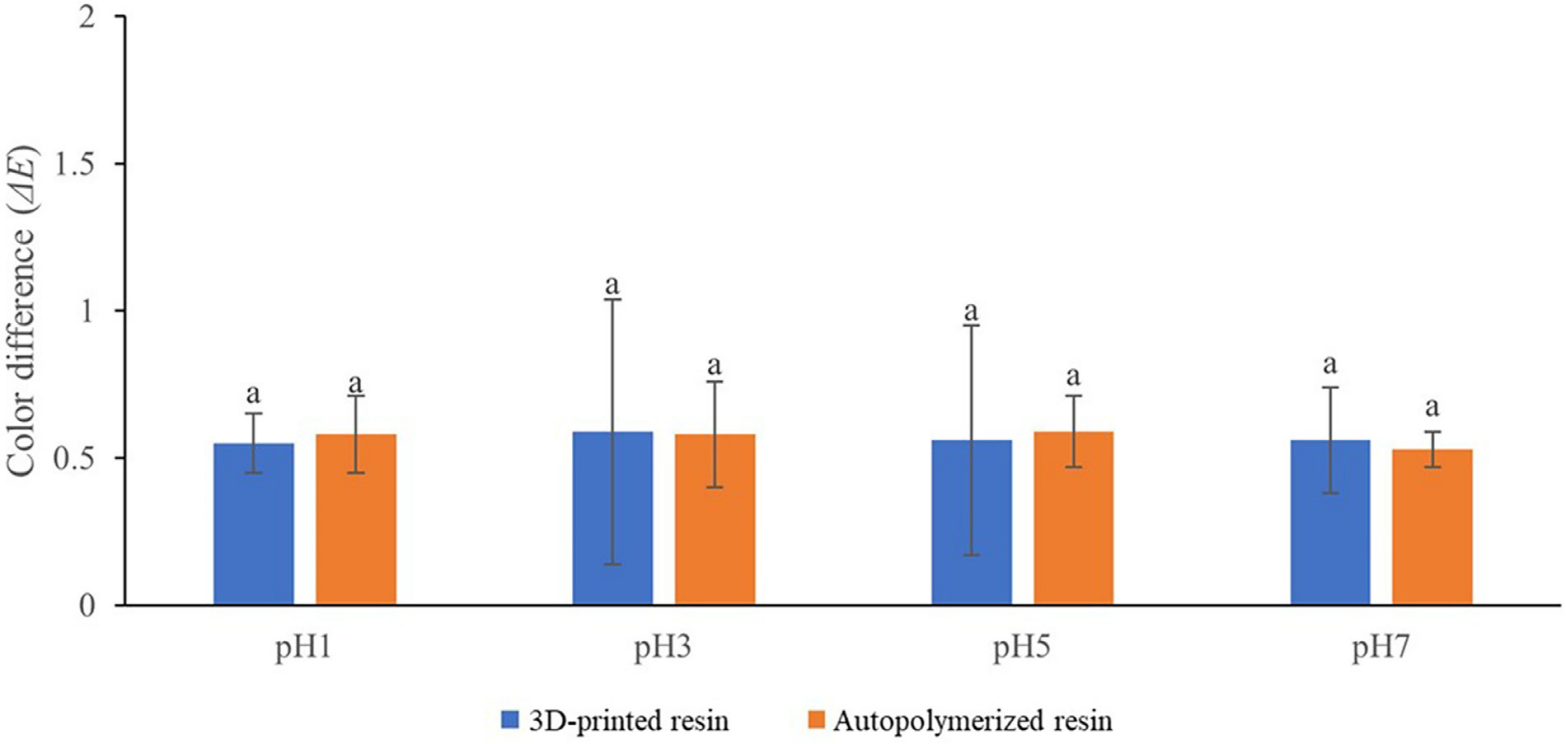
Colour changes of 3D-printed denture base resin and autopolymerized resin at different pH; same lowercase letters in the same material indicate no significant differences between pH groups (*P* > .05).

The colour difference (ΔE*) values measured before and after immersion are presented in Table and Figure 3. Across all pH levels (1, 3, 5, and 7), no statistically significant differences were observed in the colour change for either resin type (*P* > .05). Additionally, no significant differences in ΔE* were found between 3D-printed and autopolymerized resins in the same pH group (*P* > .05).

**Table:** Colour values of the 3D-printed resin and autopolymerized resin before and after immersion.

Immersion	Groups		L*	a*	b*
Before	3D-printed resin	pH 1	36.56 ± 0.56^b^	6.61 ± 0.37^a^	−0.41 ± 0.61^a^
		pH 3	36.92 ± 0.71^ab^	6.41 ± 0.16^a^	−0.55 ± 0.60^a^
		pH 5	36.26 ± 0.72^ab^	6.57 ± 0.19^a^	−0.21 ± 0.82^a^
		pH 7	35.54 ± 0.94^a^	6.69 ± 0.32^a^	−0.60 ± 0.72^a^
	Autopolymerized resin	pH 1	43.59 ± 2.38^a^	10.88 ± 0.92^a^	2.82 ± 1.00^a^
		pH 3	43.29 ± 1.57^a^	10.29 ± 1.05^a^	2.36 ± 0.91^a^
		pH 5	43.62 ± 0.88^a^	10.86 ± 0.64^a^	2.72 ± 0.74^a^
		pH 7	42.47 ± 0.72^a^	10.55 ± 0.56^a^	2.25 ± 0.36^a^
After	3D-printed resin	pH 1	36.83 ± 0.57^b^	6.41± 0.39^a^	−0.02 ± 0.58^a^
		pH 3	37.25 ± 0.51^b^	6.38 ± 0.17^a^	−0.63 ± 0.64^a^
		pH 5	36.78 ± 0.66^b^	6.41 ± 0.32^a^	−0.15 ± 0.74^a^
		pH 7	35.83 ± 0.85^a^	6.53 ± 0.33^a^	−0.52 ± 0.95^a^
	Autopolymerized resin	pH 1	43.43 ± 2.31^a^	10.91 ± 0.94^a^	2.78 ± 0.97^a^
		pH 3	43.27 ± 1.71^a^	10.46 ± 1.10^a^	2.49 ± 0.96^a^
		pH 5	43.83 ± 0.91^a^	10.61 ± 0.67^a^	2.84 ± 0.71^a^
		pH 7	42.46 ± 0.72^a^	10.44 ± 0.57^a^	2.20 ± 0.38^a^

Different lowercase letters indicate statistically significant differences among pH groups within the same material and immersion condition (*P* < .05).

### Surface roughness

**Fig. 4:**
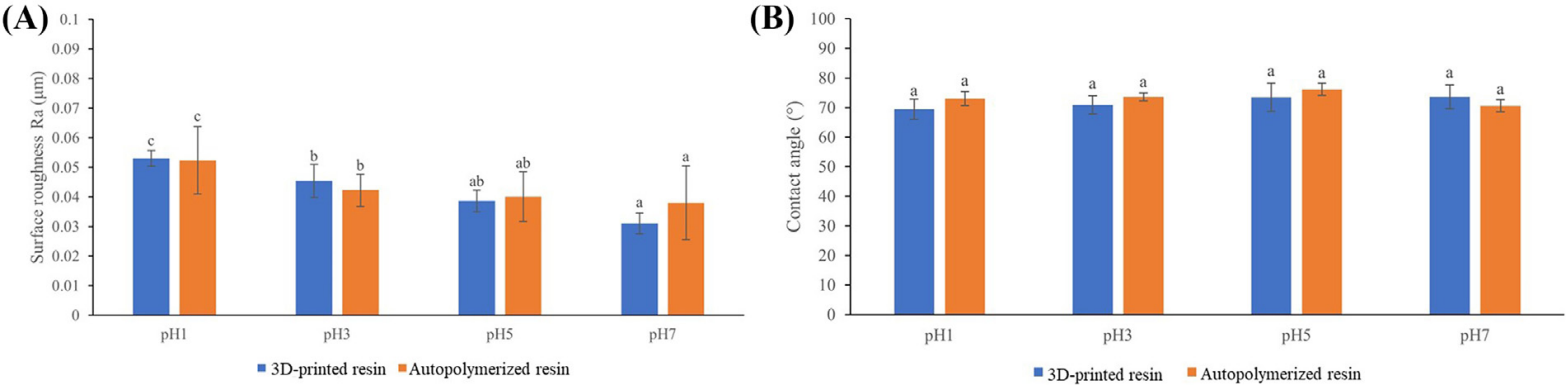
3D-printed denture base resin and autopolymerized resin by pH: (A) surface roughness and (B) contact angle. Different lowercase letters within the same material indicate significant differences among pH groups based on posthoc comparisons (*P* < .05).

Regarding surface roughness (Ra), two-way ANOVA revealed a significant main effect of pH level (*F* = 16.984, *P* < .001). However, the main effect of material type (*F* = 0.378, *P* = .541) and the interaction effect (material × pH) were not statistically significant (*F* = 1.337, *P* = .272). These results indicate that surface roughness varied according to pH level, whereas the overall roughness values did not differ significantly between the two resin types. As shown in Figure 4A, surface roughness increased under lower pH conditions in both materials. Posthoc comparisons showed that, in the 3D-printed resin group, Ra values were significantly higher at pH 1 and pH 3 than at pH 7 (*P* < .05). In the autopolymerized resin group, Ra was significantly higher at pH 1 than at pH 7 (*P* < .05).

### Contact angle

Regarding contact angle, two-way ANOVA revealed no statistically significant main effects for material type (*F* = 1.777, *P* = .194) or pH level (*F* = 1.903, *P* = .154). The interaction effect (material × pH) was also not statistically significant (*F* = 1.631, *P* = .206). These findings indicate that contact angle did not significantly differ according to material type or pH level under the tested conditions (Figure 4B).

### Surface morphology

**Fig. 5:**
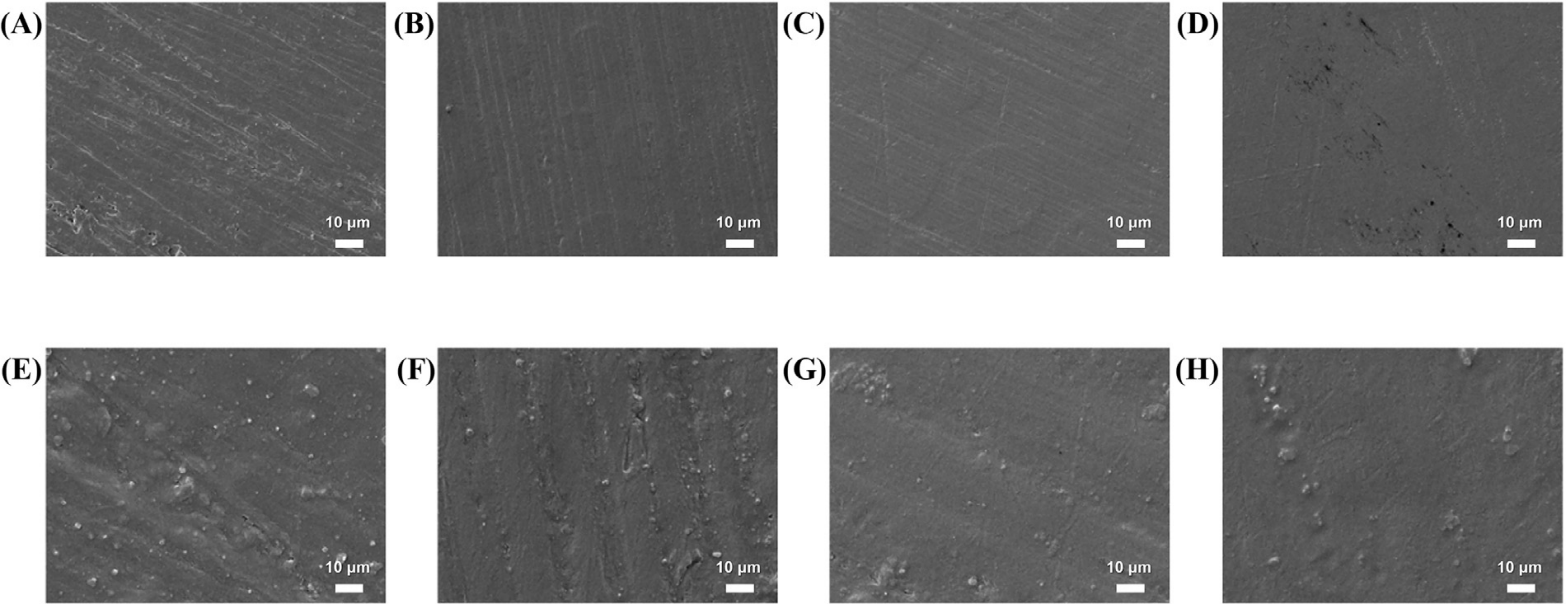
Representative scanning electron microscopy images of 3D-printed denture resin and autopolymerized resin surfaces at different pH at 5000× magnification: (A) pH 1 3D-printed resin, (B) pH 3 3D-printed resin, (C) pH 5 3D-printed resin, (D) pH 7 3D-printed resin, (E) pH 1 autopolymerized resin, (F) pH 3 autopolymerized resin, (G) pH 5 autopolymerized resin, (H) pH 7 autopolymerized resin.

The surface morphologies of the specimens at 5000× magnification are shown in Figure 5. SEM images showed pH-dependent surface morphological changes in both resin groups. In the 3D-printed resin group, specimens immersed at lower pH levels showed visible surface scratches and localized irregularities, whereas the pH 7 specimen showed a relatively smoother surface. In the autopolymerized resin group, localized surface irregularities and defect-like features were observed across the tested pH conditions. Overall, lower pH conditions were associated with more visible surface irregularities in both materials.

## Discussion

With recent advancements in digital dental technology, the use of 3D-printing methods for denture fabrication has significantly increased. Among the various additive manufacturing technologies, DLP stands out because of its capability to cure photopolymer resins layer-by-layer using high-resolution digital light sources, thereby offering precise, rapid, and smooth surface finishes compared to traditional Stereolithography Apparatus methods.^3^^,^^17^^,^^18^ DLP allows all layers to polymerize simultaneously, overcoming the inherent speed limitations of conventional Stereolithography Apparatus, and thus increasing the clinical efficiency and prosthesis accuracy.^19^^,^^20^

As 3D-printed resins have begun to gain attention as denture materials, various studies have compared them with traditional autopolymerized resins. A previous study compared the flexural strength, elastic modulus, dynamic mechanical analysis, changes in physical properties before and after thermal cycling, and surface characteristics using SEM for each material under oral conditions simulated by thermal cycling.^21^ Another study evaluated the mechanical properties and moisture absorption and solubility to compare the durability and stability of the two resins, and the results indicated that 3D-printed resins exhibit more favourable mechanical properties than conventional denture resin.^22^ This study simulated various acidic conditions in addition to the thermal cycling conditions used in previous studies and comprehensively analysed various mechanical and surface properties, such as Vickers hardness, colour stability, and contact angle, which were not included in previous studies.^11^^,^^21^

The oral environment is subject to continuous changes in acidity due to dietary habits, beverage consumption, and medication use. In particular, acidic environments can cause changes in the surface structure and strength of dental materials. In one study, 3D-printed and thermosetting resins were immersed in pH 5, 7, and 8 conditions for 3 months. The results showed that acidic conditions increased the surface roughness and hydrophilicity of the 3D-printed resins, whereas alkaline conditions tended to increase the surface roughness and hardness of the thermosetting resins. In terms of cell survival rate, 3D-printed resin showed a relatively low cell survival rate and higher cytotoxicity compared to thermosetting resin. These findings suggest that material properties may vary according to oral pH conditions and should be considered when selecting denture base materials for patients exposed to acidic challenges.^23^

This study evaluated the effects of acidic environments on the mechanical and surface properties of 3D-printed and traditional autopolymerized denture resins. Specimens were immersed in HCl-based solutions at different pH levels to evaluate the short-term response of the tested denture base resins to a controlled severe acidic challenge.^11^ These findings demonstrated that the 3D-printed resin exhibited higher flexural strength and hardness than the tested autopolymerized resin under the tested conditions. This more favourable mechanical behaviour may be attributed to the structural characteristics of DLP-printed resins, including higher polymerization density, fewer residual monomers, and reduced microporosity.^24^, ^25^, ^26^ Previous studies have similarly reported that photopolymerized materials, owing to their uniform and highly crosslinked polymeric networks, display improved mechanical stability under acidic conditions.^1^ In particular, two-way ANOVA revealed a significant interaction effect between the material type and pH level for both flexural modulus and Vickers hardness (*P* = .008). This interaction suggests that the pH-associated changes in mechanical properties may differ according to the polymer network of each resin type. Thus, the higher strength and hardness observed in the tested 3D-printed resin may reflect its material characteristics under the present experimental conditions. The different behaviours of the two materials may be explained, at least in part, by their polymer chemistry. DLP-printed denture base resins are typically based on multifunctional methacrylate monomers, including urethane dimethacrylate-related components, and undergo light-induced polymerization followed by postcuring, which may result in a highly cross-linked network and relatively high degree of conversion. This structure may reduce residual monomer content and limit acid or water penetration, thereby improving resistance to plasticization and acid-associated surface changes. Conversely, autopolymerized PMMA-based resins are formed mainly through chemical polymerization of methyl methacrylate and may contain a more heterogeneous polymer matrix with higher residual monomer content, which can increase susceptibility to water or acid uptake and matrix softening. These chemical and structural differences may partly explain the lower mechanical properties and more visible surface irregularities observed in the autopolymerized resin. However, because chemical analyses were not performed, the SEM findings should be interpreted as qualitative morphological evidence rather than direct confirmation of polymer matrix hydrolysis.

Regarding surface roughness (Ra), two-way ANOVA demonstrated a significant main effect of pH level (*P* < .001), whereas material type and the material × pH interaction were not statistically significant. These results indicate that Ra was mainly associated with pH level, while the pH-associated change did not differ significantly between the two tested materials. SEM was used as a qualitative method to observe surface morphology, whereas surface roughness was quantitatively evaluated using stylus profilometry and expressed as Ra values. Clinically, a surface roughness threshold of 0.2 μm has been commonly cited as a reference value associated with increased bacterial adhesion and biofilm accumulation. In this study, the measured Ra values remained below this threshold across the tested pH groups; however, this finding should be interpreted with caution because biofilm formation was not directly evaluated. In addition, although surface roughness was measured in different directions to reduce directional measurement bias, stylus profilometry remains a line-based method and may not fully capture wide-area 3D surface topography. Higher-resolution and wider-area surface analyses, such as confocal microscopy, atomic force microscopy, optical profilometry, or 3D surface mapping, were not performed, which limits the ability to fully characterize micro- or nano-scale surface alterations. Therefore, the SEM observations should be considered qualitative visual support for the profilometric roughness results rather than quantitative evidence of microscale surface changes. The more visible surface features observed in the autopolymerized resin may be related to differences in polymerization mechanisms and material homogeneity; however, because quantitative image analysis was not performed, these morphological observations should be interpreted cautiously. Increased surface roughness may have clinical implications because rougher surfaces can facilitate biofilm accumulation, bacterial colonization, and staining susceptibility. In addition, recent evidence on monolithic zirconia has shown that surface treatment–induced changes can influence bonding performance at the material–dentin interface, further supporting the broader importance of surface characteristics in the clinical behaviour of dental materials.^27^^,^^28^

Despite differences in mechanical properties and qualitative surface morphology, both materials exhibited similar colour stability and contact angle behaviour under all tested pH conditions. Short-term acidic exposure appeared to be insufficient to induce significant colour changes or wettability alterations, which agrees with earlier findings by Moon et al.^29^^,^^30^ The limited colour changes observed in the tested materials may be related to the short-term immersion period and the relatively stable polymeric matrices, although further long-term ageing studies are required to confirm their optical stability.^25^^,^^31^

Despite these findings, several limitations of this study should be acknowledged. First, the comparison was limited to two specific commercial resins, one DLP-printed denture base resin and one autopolymerized denture base resin, and therefore the findings may not represent the broad range of 3D-printed, milled, heat-polymerized, and autopolymerized denture base materials currently available. Second, a major limitation of this study is the exclusion of heat-polymerized PMMA, which is universally considered the gold standard for definitive denture bases. Since autopolymerized resins often exhibit inferior mechanical properties to heat-cured standards, the more favourable mechanical properties observed for the tested 3D-printed resin should be interpreted within this specific comparative context. Regarding the immersion protocol, the 24 hours immersion in HCl was designed to represent an acute acidic challenge – specifically, a worst-case scenario of gastric acid exposure – rather than chronic, long-term exposure. While HCl is the primary acidic component of gastric juice, this simplified immersion model does not account for the intermittent nature of clinical acid exposure, saliva buffering, salivary pellicle formation, thermal fluctuations, or biofilm interactions. In addition, because the acidic challenge was limited to HCl, this model does not fully reflect the broad spectrum of acidic exposures encountered in the oral cavity, including dietary organic acids, acidic beverages, and bacterial acid production. Therefore, the short immersion period should be considered a significant limitation, and the present findings should not be interpreted as evidence of long-term clinical durability. Further investigations incorporating accelerated artificial ageing, thermocycling, repeated cyclic acidic exposure, and saliva- or pellicle-containing models are necessary to evaluate long-term clinical performance. Another limitation is that baseline measurements before immersion or unimmersed control specimens were not included for flexural strength, flexural modulus, Vickers hardness, and surface roughness. Therefore, true percentage degradation or degradation rates could not be calculated for these properties. Although the pH 7 group served as a neutral immersion control, it was not equivalent to an unimmersed baseline. Accordingly, the present findings should be interpreted as pH-associated differences after short-term immersion rather than as direct evidence of degradation from the original material state. Future studies should include unimmersed baseline controls and calculate percentage changes after clinically relevant ageing protocols. Third, statistically significant baseline differences in preimmersion L* values were observed among the 3D-printed resin subgroups (Table), indicating incomplete baseline optical standardization before acidic immersion. These differences may have resulted from subtle variations in postprinting processing, polishing, or initial surface conditions. Therefore, the absolute colour-coordinate results should be interpreted with caution. To reduce the influence of these baseline discrepancies, this study focused on relative colour changes (ΔE*) rather than absolute coordinates. Additionally, although surface roughness was measured in different directions to reduce directional measurement bias, stylus profilometry remains a line-based method and may not fully capture wide-area 3D surface topography. Its 2 μm probe tip may also have limited resolution for detecting fine microscale features near the 0.2 μm threshold, potentially underestimating surface topography relevant to biofilm accumulation. Future studies using wider-area and higher-resolution surface analysis methods, such as confocal microscopy, optical profilometry, atomic force microscopy, or 3D surface mapping, are warranted.

In summary, the tested 3D-printed resin showed more favourable mechanical properties than the tested autopolymerized resin after short-term immersion under the tested acidic conditions. However, because this study evaluated only two commercial materials and did not include unimmersed baseline controls or long-term ageing protocols, the findings should be interpreted cautiously. Further studies involving broader material formulations, baseline measurements, repeated acidic ageing, and clinically relevant oral simulation models are required.

## Conclusion

Within the limitations of this *in vitro* study, the tested 3D-printed resin (NextDent Denture 3D+) exhibited more favourable mechanical properties than the tested autopolymerized resin (ProBase Cold) after short-term immersion under the tested pH conditions. Both materials showed increased surface roughness under lower pH conditions and minimal colour change across the tested pH levels. However, because unimmersed baseline controls were not included, these findings should be interpreted as short-term pH-associated differences between the two tested commercial materials rather than as direct evidence of degradation rate or long-term clinical durability. Further studies incorporating baseline measurements, repeated acidic ageing protocols, clinically relevant oral simulation models, and a broader range of denture base materials are warranted.

## Data availability

The datasets used and analysed during the current study are available from the corresponding author on reasonable request.

## Author contributions

Eun-Ju Kim: Writing – original draft, methodology. Se-Eun Kim: Writing – original draft, investigation, formal analysis. Ye-Jin Kim: Methodology, formal analysis. Ji-Eun Kim: Writing – original draft, resources. Yun-Yeong Hwang: Methodology, investigation, resources. Hye-Bin Go: Writing – review and editing, supervision, validation. Song-Yi Yang: Writing – review and editing, supervision, project administration.

## Conflict of interest

The authors declare that they have no known competing financial interests or personal relationships that could have appeared to influence the work reported in this article.
